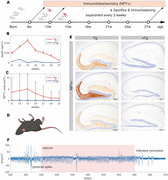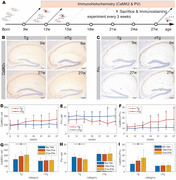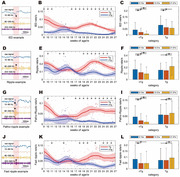# Temporal Progression of Pathophysiological Changes in hAPP‐J20 Transgenic Alzheimer’s Mice: Insights from Longitudinal Analysis

**DOI:** 10.1002/alz.087939

**Published:** 2025-01-03

**Authors:** Yue Loong Hsin, Keng Ying Liao, Xu Han, Wen‐Ying Chen, Wentai Liu

**Affiliations:** ^1^ Department of Neurology, Chung Shan Medical University Hospital, Taichung, Taichung Taiwan; ^2^ Department of Veterinary Medicine, National Chung Hsing University, Taichung Taiwan; ^3^ Department of Bioengineering, University of California, Los Angeles, CA, USA, Los Angeles, CA USA; ^4^ Department of Veterinary Medicine, National Chung Hsing University, Taichung, Taichung Taiwan; ^5^ UCLA California NanoSystems Institute, University of California, Los Angeles, CA, USA, Los Angeles, CA USA

## Abstract

**Background:**

Alzheimer’s disease (AD) is characterized by cognitive decline and increased seizure susceptibility due to brain damage and neural disruptions. This study examines the relationship between cognitive deterioration and seizure pathology in hAPP‐J20 transgenic Alzheimer’s mice, a model known for amyloid plaque deposition and heightened seizure activity.

**Method:**

We observed hAPP‐J20 mice aged 8 to 28 weeks using long‐term wireless telemetry to assess hippocampal local field potential, sampled at 2 kHz. This allowed categorization of high‐frequency oscillations (HFOs) into ripples (80‐200 Hz) and fast ripples (250‐600 Hz). We conducted the Morris water maze test for spatial learning assessment, analyzed amyloid plaque deposition, seizure‐related deaths, neuropeptide Y (NPY) concentration, quantified glutamatergic pyramidal cells and GABAergic parvalbumin (PV) interneurons, and measured the excitatory/inhibitory cell ratio.

**Result:**

Amyloid plaque deposition commenced post‐18 weeks, marking a pivotal phase in disease progression. This period correlated with a peak in seizure‐related deaths, which reached its zenith at 15 weeks and subsequently declined gradually. In parallel, the NPY concentration surged, peaking concurrently with the peak in seizure‐related deaths at 15 weeks before tapering off. Meanwhile, the count of pyramidal cells showed a mild upward trend. In contrast, the population of PV interneurons demonstrated a discernible decrease starting from 15 weeks, with a significant reduction post‐18 weeks, aligning with the initiation of amyloid plaque staining in the hippocampus. The ratio of excitatory to inhibitory cells began increasing from 9 weeks, peaking after 15 weeks. The rates of HFOs, encompassing both ripples and fast ripples, exhibited an upward trajectory preceding the 18‐week mark, reflecting an increase in neural network excitability during this critical period.

**Conclusion:**

This study outlines the progression of pathophysiological changes in the AD mouse model, linking amyloid plaque deposition to seizure activity, NPY levels, neuronal cell density changes, and increased neural network excitability. The rise in HFO rates, especially fast ripples, suggests their potential as biomarkers for seizure onset or epileptogenesis, linking amyloid pathology with seizure pathology. These findings provide crucial insights for the development and timing of therapeutic strategies in AD.